# Anxiety and depression due to 2019 SARS-CoV-2 among frontier healthcare workers in Kenya

**DOI:** 10.1016/j.heliyon.2021.e06351

**Published:** 2021-02-23

**Authors:** David Onchonga, Enoch Ngetich, Wilbroda Makunda, Pius Wainaina, Diana Wangeshi, Prémusz viktoria

**Affiliations:** a Doctoral School of Health Sciences, Faculty of Health Sciences, University of Pécs, Hungary; b School of Public health, Mount Kenya University, Nairobi, Kenya; c Ministry of Health, Kisumu, Kenya; d Technical University of Kenya, Nairobi, Kenya; e Debrecen University, Hungary

**Keywords:** Generalized anxiety disorder scale, Patient health questionnaire, Anxiety and depression scale, Mental health, Coronavirus

## Abstract

**Background:**

The novel coronavirus disease continues to spread across the globe, causing anxiety and depression among healthcare workers.

**Objectives:**

The current study aimed to determine the levels of anxiety and depression due to the coronavirus pandemic among healthcare workers in Kenya.

**Methods:**

A total sample of 476 respondents participated. The 7-item Generalized Anxiety Disorder Scale (GAD-7) and Patient-Health Questionnaire (PHQ-9), together with a socio-demographic questionnaire, were applied. Stratified sampling was used. Data was analysed using the Statistical Package Programme for Social Science Version 23.0.0. Kruskal Wallis test and Mann-Whitney U test were employed to establish the differences in levels of anxiety and depression across socio-demographic characteristics. Ordinal logistic regression analysis was used to establish the predictors of levels of anxiety and depression, and associations were considered significant at *p* < 0.05.

**Results:**

A total of 35.1% (n = 167) of the participants had mild anxiety, and 13.4% (n = 64) severe anxiety. Approximately 53.6% (n = 255) had mild depression while 9.2% (n = 44) had severe depression. The univariate analysis illustrated a statistical difference in anxiety levels in gender (*p* > 0.027), years of work experience (*p* = 0.005), and the cadre of respondents (*p* = 0.0028). Gender was statistically significant with the level of depression (*p* = 0.045). About 62.6% (n = 298) of healthcare workers had been trained, and only 9% (n = 43) were confident in managing COVID-19 cases. A large proportion, 98% (n = 458) had concerns about the availability of personal protective equipment.

**Conclusion:**

The study findings indicated that the majority of healthcare workers had mild anxiety and depression. Female healthcare workers were more likely to experience severe anxiety and depression. Also, levels of anxiety and depression differed across different cadres of healthcare workers.

## 1 Introduction

Coronaviruses are a group of viruses belonging to the family of Coronaviridae, which infect both animals and humans [1]. Human coronaviruses can cause a mild disease similar to a common cold, while others cause severe diseases (Middle East Respiratory Syndrome and Severe Acute Respiratory Syndrome). Previously not identified in humans, a new coronavirus emerged in Wuhan, China in December 2019 [2], and within three months, the virus had spread across all continents [2, 3, 4, 5, 6, 7]. Considering its global threat, the World Health Organization (WHO) on January 30^th^, 2020 declared it a public health emergency of global concern [1, 8]. According to the WHO coronavirus Disease (COVID-19) dashboard posted on 13^th^ February 2021 [9], there were 107,686,655 confirmed cases of COVID-19 disease with 2,368,571 deaths globally. Continuously, numerous countries are recording a large number of cases daily. Western Pacific has 1,524,130 cases, Europe 36,294,484 cases, South-Eastern Asia 13,165,612 cases, Eastern Mediterranean region 5,975,060 cases, the Americas 48,021,725 cases, and the African region 2,703,899 cases. In that regard, panic among members of the public is imminent, which increases mental stress and excruciating psychological distress [10].

Coronaviruses (CoVs) belong to a family of viruses responsible to cause respiratory infection in humans and enteritis in animals [11]. The 2019-nCoV (now commonly known as COVID-19) is a new coronavirus that has not been previously identified [12]. Most patients show mild to moderate, pneumonia-like symptoms, and may recover without special treatment [13]. However, older people and those with underlying medical conditions develop a severe acute respiratory infection. The initial case of COVID-19 disease demonstrated a pneumonia case of unknown cause after being assessed for viral pneumonia by testing of bronchoalveolar-lavage fluid using complete genome sequencing, cell cultures, and polymerase chain reaction (PCR) [14, 15, 16, 17, 18, 19].

The current COVID-19 disease has an evidently higher mortality rates in adults, unlike the human rhinoviruses and human adenoviruses which are prevalent in children [20, 21]. Frontier healthcare workers are mandated to control and manage epidemics based on their professional oaths of conduct and codes of regulation. Due to it's high infection rate, the WHO has recommended strategic infection prevention and control (IPC) measures among healthcare workers [22]. Ideally, these would reduce the rate of infection considering their high vulnerability to the virus [23, 24, 25, 26]. Furthermore, the public expects healthcare workers to reduce the burden amid the crisis. However, the crisis may be severe in developing countries where most health facilities are challenged with inadequate staffing, medical facilities, medical supplies, diagnostic equipment, and reagents. In that regard, the unprecedented influx of COVID-19 cases may further stretch the already inadequate human resource for health. Consequently, the healthcare workers may be working for long hours in stressful environments, which may lead to fatigue, anxiety, and depression. In China, a psychological impact report of the COVID-19 pandemic on the healthcare workers and the general public has provided insightful facts. Therefore, healthcare workers in developing countries like Kenya should expect similar effects and perhaps severe due to other challenges such as inadequate budgetary allocation to healthcare services, out-of-pocket spending for healthcare services, inadequacies in healthcare infrastructure and systemic challenges due to challenges in implementation of key policies set by the government [27, 28].

Although there have been studies on the prevalence of self-medication among healthcare workers [29] and the general public [30] before and during the COVID-19 pandemic attributed to the current pandemic, to the best of our knowledge and by the time of publication of this manuscript, there are no detailed studies on the levels of anxiety and depression among healthcare workers in Kenya due to the current COVID-19 pandemic. Therefore, this study aimed to evaluate the state of anxiety and depression due to the novel 2019 coronavirus disease among frontier healthcare workers in Kenya.

## 2 Materials and methods

### 2.1 Study design

A cross-sectional study design was employed in this study among 476 frontier healthcare workers actively involved in the management of the COVID-19 disease pandemic. Stratified sampling was used in this study. Firstly, different cadres of frontier healthcare workers were used as strata and in each stratum, an online questionnaire was sent to the healthcare professional online groups and platforms such as WhatsApp and Facebook. From each stratum, data was collected through random sampling of the received questionnaires until the required sample was reached. The shared questionnaire was anonymous to ensure data confidentiality and reliability.

### 2.2 Sample size determination

The sample size was determined by using the following formula: N = Z_α_^2^ P (1−P)/d^2^, where α was 0.05, Z_α_ was 1.96 (at the 95% confidence level) and the estimated acceptable margin of error for proportion d was 0.05. The minimum sample would be 385 participants but we ended up recruiting 476 participants.

### 2.3 Data collection tools

The study adopted two validated questionnaires; the Generalized Anxiety Disorder Scale (GAD-7) to assess the level of anxiety and a Patient Health Questionnaire (PHQ-9) to assess the level of depression. A social-demographic section was added to capture information on; gender, age, level of education, years of experience, marital status, religion, the cluster of health facilities deployed, cadre, and on-job-training on COVID-19 disease.

The Generalized Anxiety Disorder (GAD-7) questionnaire is a validated seven-item, self-report anxiety questionnaire designed by Spitzer *et al.* [31] to assess the patient's health status within two (2) weeks. The items indicate the level an individual feels nervous, anxious or on edge, unable to control worrying, worrying too much about different things, having trouble relaxing, being restless, being irritable, and being afraid that something negative might happen. Scores from 0, 1, 2, or 3 were tagged on symptoms; ‘not at all’, ‘several days’, ‘more than half the days,’ and ‘nearly every day’, respectively. Then, the total scoresare presented from 0 to 21. Scores 5, 10, and 15 represented cut-off points for mild, moderate, and severe anxiety respectively [32, 33, 34]. When screening for an anxiety disorder, a score of greater than 10 is recommended for further evaluation [35].

The PHQ-9 is widely used as an open-access screening instrument for depression in different healthcare and community settings [36]. The instrument tallies nine questions from "0" (not at all) to "3" (nearly every day). The tool was developed as a fully self-administered version of the original PRIME-MD by Spitzer *et al.* [37]*.* PHQ-9 total scores for the nine items range from 0 to 27. The nine items include the experience of pleasure, feeling down, sleep disruption, energy levels, appetite, feeling of failure, trouble concentrating, speaking slowly or being fidgety, and having suicidal thoughts or self-harm over the past 2 weeks. The tool has been validated for use in primary care. Furthermore, aspects of the construct validity of the PHQ-9 have been documented in studies done both in medical settings and in the general population, exhibiting strong associations of the PHQ-9 depression severity score with diverse aspects of health-related quality of life [36, 38, 39].

### 2.4 Data analysis and presentation

Data was analysed using the Statistical Package Programme for Social Science Version 23.0.0 by IBM. Kruskal Wallis test and Mann-Whitney U test were used to establish the differences in levels of anxiety and depression across socio-demographic characteristics. Ordinal logistic regression analysis was applied to establish the predictors of the levels of anxiety and depression among healthcare workers and associations were considered significant at *p* < 0.05.

### 2.5 Ethical approvals

The approval to conduct this research was obtained from the Jaramogi Oginga Odinga Teaching and Referral Hospital Ethical Review Committee (IERC/JOOTRH/209/20). All the respondents were informed of the objectives of the study and were required to consent before enrolment into the study.

## 3 Results

Among the 476 respondents, half were female, the majority aged between 31-40 years old, and 45.6% (n = 217) had a bachelor's degree. Slightly above a third of respondents (37.8%) had worked for not more than five years. A large proportion of the healthcare workers were married (63.2%), Christians by religion (92.2%), and worked in County and Sub-county hospitals as shown in Table 1. About 62.6% (=298) of respondents in the study at the time of data collection had been trained on COVID-19 while 37.4% (n = 37.4) had not.

**Table 1:** Socio-demographic characteristics of respondents.

Variable	Frequency (n)	Percent (%)
**Gender**		
Male	231	48.5
Female	245	51.5
Total	476	100.0
**Age**		
18–30	175	36.8
31–40	191	40.1
41–50	77	16.2
above 51	33	6.9
Total	476	100.0
**Level of education**		
certificate	17	3.6
diploma	196	41.2
Higher diploma	46	9.7
Degree	217	45.6
Total	476	100.0
**Years of experience**		
0–5 yrs.	180	37.8
6–10 yrs.	130	27.3
above 11 yrs.	166	34.9
Total	476	100.0
**Marital status**		
single	175	36.8
married	301	63.2
Total	476	100.0
**Religion**		
Christian	439	92.2
Muslim	32	6.7
others	5	1.1
Total	476	100.0
**Place of work**		
dispensary	47	9.9
health Centre	60	12.6
county and sub-county hospital	188	39.5
administration	50	10.5
private health facility	78	16.4
Referral hospitals including university hospitals	53	11.1
Total	476	100.0

### 3.1 Level of anxiety

A majority of the healthcare workers in the survey had mild anxiety about the COVID-19 disease pandemic, 33.8% (n = 161) had moderate anxiety while 17.6% (n = 84) had moderately severe anxiety, and 13.4% (n = 64) had exhibited severe anxiety as shown in Table 2.

**Table 2:** Levels of anxiety of respondents.

Anxiety Level	Frequency (n)	Percent (%)
Mild Anxiety	167	35.1
Moderate anxiety	161	33.8
Moderately severe anxiety	84	17.6
Severe anxiety	64	13.4
Total	476	100.0

### 3.2 Level of depression

Approximately 53.6% (n = 255) of healthcare workers had a mild form of depression, 26.9% (n = 128) moderate depression, 10.3% (n = 49) moderately severe depression, and 9.2% (n = 44) severe depression as indicated in Table 3.

**Table 3:** Levels of depression of respondents.

Depression Level	Frequency (n)	Percent (%)
Mild depression	255	53.6
Moderate depression	128	26.9
Moderately severe depression	49	10.3
Severe depression	44	9.2
Total	476	100.0

### 3.3 Factors influencing frontier healthcare workers anxiety

As shown in Table 4, the univariate analysis illustrated a statistical difference in anxiety levels and the respondent's gender (*p* > 0.027), years of work experience, (*p = 0.005*), and the cadre (*p = 0.0028*). Univariate analysis of the association between socio-demographic characteristics and levels of depression had a statistical difference based on respondents' genders and the level of depression due to the COVID-19 disease pandemic (*p* = *0.045).* There were no statistically significant differences across other socio-demographic characteristics based on the level of depression as shown in Table 5.

**Table 4:** Univariate analysis of anxiety levels across sociodemographic variables.

Variable	Levels of Anxiety				Total	Statistics	p-values
	Mild anxiety	Moderate anxiety	Moderately severe anxiety	Severe Anxiety			
**Gender**							
Male	93 (40.3%)	73 (31.6%)	41 (17.7%)	24 (10.4%)	231	4.897^a^	0.027
Female	74 (30.2%)	88 (35.9%)	43 (17.6%)	40 (16.3%)	245		

**Age**							
18–30	67 (38.3%)	53 (30.3%)	35 (20%)	20 (11.4%)	175	1.816^b^	0.612
31–40	68 (35.6%)	62 (32.5%)	29 (15.2%)	32 (16.8%)	191		
41–50	19 (24.7%)	34 (44.2%)	16 (20.8%)	8 (10.4%)	77		
Above 51	13 (39.4%)	12 (36.4%)	4 (12.1%)	4 (12.1%)	33		
**Level of Education**							
Certificate	8 (47.1%)	3 (17.6%)	3 (17.6%)	3 (17.6%)	17	7.812^b^	0.553
Diploma	73 (37.2%)	71 (36.2%)	30 (15.3%)	22 (11.2%)	196		
Higher Diploma	16 (34.8%)	18 (39.1%)	8 (17.4%)	4 (8.7%)	46		
Degree	70 (32.3%)	69 (31.8%)	43 (19.8%)	35 (16.1%)	217		
**Years of Experience**							
0-5 Yrs.	77 (42.8%)	51 (28.3%)	37 (20.6%)	15 (8.3%)	180	5.912^b^	0.005
6-10 Yrs.	40 (30.8%)	45 (34.6%)	18 (13.8%)	27 (20.8%)	130		
Above 11 Yrs.	50 (30.1%)	65 (39.2%)	29 (17.5%)	22 (13.3%)	166		
**Marital Status**							
Single	72 (41.1%)	56 (32%)	26 (14.9%)	21 (12%)	175	4.818^a^	0.186
Married	95 (31.6%)	105 (34.9%)	58 (19.3%)	43 (14.3%)	301		
**Religion**							
Christian	159 (36.2%)	153 (34.9%)	73 (16.6%)	54 (12.3%)	439	14.575^b^	0.024
Muslim	6 (18.8%)	8 (25%)	9 (28.1%)	9 (28.1%)	32		
Others	2 (40%)	0 (0%)	2 (40%)	1 (20%)	5		
**Place of Work**							
Dispensary	14 (29.8%)	19 (40.4%0	7 (14.9%)	7 (14.9%)	47	1.1^b^	0.9541
Health Centre	19 (31.7%)	23 (38.3%)	14 (23.3%)	4 (6.7%)	60		
County &Sub County Hospital	70 (37.2%)	65 (34.6%)	29 (15.4%)	24 (12.8%)	188		
Administration	16 (32%)	16 (32%)	14 (28%)	4 (8%)	50		
Private Health Facility	29 (37.2%)	21 (26.9%)	13 (16.7%)	15 (19.2%)	78		
Referral & university hospitals	19 (35.8%)	17 (32.1%)	7 (13.2%)	10 (18.9%)	53		
**Cadre**							
Administration	14 (42.4%)	8 (24.2%)	11 (33.3%)	0 (0%)	33	21.711^b^	0.0028
Public Health	28 (36.8%)	26 (34.2%)	11 (14.5%)	11 (14.5%)	76		
Nursing Officer	56 (26.9%)	80 (38.5%)	37 (17.8%)	35 (16.8%)	208		
Clinical Officer	24 (48%)	13 (26%)	7 (14%)	6 (12%)	50		
Medical Officer	15 (30.6%)	12 (24.5%)	11 (22.4%)	11 (22.4%)	49		
Lab Scientist and technologists	9 (42.9%)	8 (38.1%)	4 (19%)	0 (0%)	21		
Pharmacist and technologists	15 (57.7%)	11 (42.3%)	0 (0%)	0 (0%)	26		
Physiotherapists & technicians	6 (46.2%)	3 (23.1%)	3 (23.1%)	1 (7.7%)	13		

a Mann Whitney U Test.

b Kruskal Wallis Rank Sum Test.

**Table 5:** Univariate statistics for association between the sociodemographic characteristics and levels of depression.

Levels of Depression							
Variable	Mild depression	Moderate depression	Moderately severe depression	Severe depression	Total	statistic	p-values
**Gender**							
Male	136 (58.6%)	56 (24.2%)	20 (8.7%)	19 (8.2%)	231	3.995^a^	0.045
Female	119 (48.6%)	72 (29.4%)	29 (11.8%)	25 (10.2%)	245		
**Age**							
18–30	89 (50.9%)	47 (26.9%)	19 (10.9%)	20 (11.4%)	175	3.102^b^	0.3761
31–40	109 (57.1%)	50 (26.2%)	17 (8.9%)	15 (7.9%)	191		
41–50	37 (48.1%)	23 (29.9%)	9 (11.7%)	8 (10.4%)	77		
above 51	20 (60.6%)	8 (24.2%)	4 (12.1%)	1 (3%)	33		
**Level of education**							
Certificate	11 (64.7%)	1 (5.9%)	3 (17.6%)	2 (11.8%)	17	0.184^b^	0.9801
Diploma	101 (51.5%)	62 (31.6%)	24 (12.2%)	9 (4.6%)	196		
Higher diploma	22 (47.8%)	18 (39.1%)	2 (4.3%)	4 (8.7%)	46		
Degree	121 (55.8%)	47 (21.7%)	20 (9.2%)	29 (13.4%)	217		
**Years of experience**							
0–5 yrs.	98 (54.4%)	48 (26.7%)	20 (11.1%)	14 (7.8%)	180	1.4993^b^	0.96
6–10 yrs.	68 (52.3%)	34 (26.2%)	13 (10%)	15 (11.5%)	130		
above 11 yrs.	89 (53.6%)	46 (27.7%)	16 (9.6%)	15 (9%)	166		
**Marital status**							
single	98 (56%)	48 (27.4%)	15 (8.6%)	14 (8%)	175	1.5954^a^	0.66
married	157 (52.2%)	80 (26.6%)	34 (11.3%)	30 (10%)	301		
**Religion**							
Christian	239 (54.4%)	118 (26.9%)	43 (9.8%)	39 (8.9%)	439	5.1742^b^	0.522
Muslim	13 (40.6%)	10 (31.3%)	5 (15.6%)	4 (12.5%)	32		
others	3 (60%)	0 (0%)	1 (20%)	1 (20%)	5		
**Place of work**							
dispensary	27 (57.4%)	10 (21.3%)	3 (6.4%)	7 (14.9%)	47	7.3373^b^	0.948
health Centre	33 (55%)	17 (28.3%)	5 (8.3%)	5 (8.3%)	60		
county and sub county hospital	102 (54.3%)	49 (26.1%)	21 (11.2%)	16 (8.5%)	188		
administration	22 (44%)	17 (34%)	5 (10%)	6 (12%)	50		
private health facility	41 (52.6%)	23 (29.5%)	8 (10.3%)	6 (7.7%)	78		
referral hospitals including university hospitals	30 (56.6%)	12 (22.6%)	7 (13.2%)	4 (7.5%)	53		
**Cadre**							
administration	18 (54.5%)	9 (27.3%)	4 (12.1%)	2 (6.1%)	33	31.3084^b^	0.052
public health	45 (59.2%)	16 (21.1%)	6 (7.9%)	9 (11.8%)	76		
nursing officer	97 (46.6%)	64 (30.8%)	28 (13.5%)	19 (9.1%)	208		
clinical officer	27 (54%)	14 (28%)	3 (6%)	6 (12%)	50		
medical officer	27 (55.1%)	7 (14.3%)	8 (16.3%)	7 (14.3%)	49		
Lab scientist and technologists	12 (57.1%)	9 (42.9%)	0 (0%)	0 (0%)	21		
pharmacist and technologists	20 (76.9%)	6 (23.1%)	0 (0%)	0 (0%)	26		
Physiotherapists and technicians	9 (69.2%)	3 (23.1%)	0 (0%)	1 (7.7%)	13		

a Mann Whitney U Test.

b Kruskal Wallis Rank sum Test.

### 3.4 Ordinal logistic regression

Significant variables associated with the anxiety level included in the univariate analysis were subjected to ordinal regression analysis. Model fitting information illustrates that the final model used for regression analysis returned a significant value (χ^2^ = 52.317, DF = 24, *p* = 0.01). Parameter estimates revealed that for every unit increase in the level of anxiety; male respondents were 0.85 times less likely to experience anxiety as a result of COVID-19 than female *(p* = 0.013) as shown in Table 6.

**Table 6:** Ordinal logistic regression analysis.

Variables Total (n)	Sig	A.O.R	Lower	Upper
**Gender**				
Male 231	0.013	0.855	0.579	1.262
Female 245		1		
**Age-Groups**				
18–30 Years 175	0.094	2.325	0.867	6.236
31–40 Years 191	0.499	1.314	0.595	2.901
41–50 Years. 77	0.309	1.484	0.693	3.175
Above 51 Years. 33		1		
**Level of education**				
Certificate 17	0.349	0.626	0.234	1.669
Diploma 196	0.15	0.74	0.491	1.115
Higher Diploma 46	0.78	0.913	0.482	1.731
Degree 217		1		
**Years of Experience**				
0–5 Years. 180	0.092	0.521	0.244	1.113
6–10 Years. 130	0.421	1.265	0.714	2.241
Above 11 Years. 166		1		
**Marital Status**				
Single 175	0.181	0.747	0.486	1.146
Married 301		1		
**Religion**				
Christian 439	0.629	0.665	0.127	3.473
Muslim 32	0.575	1.657	0.284	9.679
Others 5		1		
**Workplace**				
Dispensary. 47	0.668	1.187	0.542	2.601
Health Centre. 60	0.359	1.402	0.681	2.884
County and sub county hospital. 188	0.88	1.046	0.581	1.884
Administration. 50	0.495	1.305	0.607	2.804
Private health facility. 78	0.158	1.639	0.825	3.257
Referral hospitals 53		1		
**Cadre of Respondents**				
Administration. 33	0.585	0.708	0.204	2.453
Public health 76	0.827	0.882	0.285	2.725
Nursing officer. 208	0.628	1.309	0.44	3.894
Clinical officer. 50	0.442	0.627	0.19	2.061
Medical officer. 49	0.5	1.513	0.454	5.035
Lab scientist and technologists. 21	0.672	0.75	0.199	2.834
Pharmacist and technologists. 26	0.069	0.288	0.075	1.104
Physiotherapists and technicians. 13		1		

### 3.5 The perceived confidence level of frontier healthcare workers in managing COVID-19 cases

About half of the respondents were somewhat confident in managing COVID-19, and two-fifth were not confident at all. Only 9% (n = 43) of respondents in the survey were very confident in managing COVID-19 cases as indicated in Table 7.

**Table 7:** Perceived confidence in managing COVID-19 cases.

Perceived confidence level in handling COVID-19	Frequency (n)	Percentage (%)
Very confident	43	9.0
Somehow confident	234	49.2
Not confident at all	199	41.8
Total	476	100.0

## 4 Discussion

The current study's main aim was to assess anxiety and depression due to the novel 2019 coronaviruses disease among frontier healthcare workers. The study results indicated that the majority of healthcare workers across the mentioned cadres had mild anxiety (35.1%, n = 167) while those who had moderate-severe and severe anxieties were 17.6% and 13.4% respectively. Studies have indicated that during public health emergencies, frontier healthcare workers can experience numerous psychological effects, which can manifest as anxiety, fear, and panic, especially in highly impactful outbreaks such as COVID-19 disease [40, 41]. This would be a result of the novelty of the disease and the fact that frontier healthcare workers are the first line of response in such situations.

In the current study, gender, years of experience, and the cadre of respondents were statistically significant in terms of anxiety. Female participants were more anxious compared to their male counterparts, and the impact on nurses was severe compared to other cadres. The current study agrees with a similar study that has shown similar results [41]. Also, studies have attributed this to the level of interactions and roles played by women in society. Therefore, their duties at the health facilities may predispose them to the virus, which would endanger their families at home [42].

Nurses are the first frontline healthcare workers to interact with patients testing positive, which makes them more vulnerable. This not only poses a danger to them but also their peers, family members, and relatives with whom they interact and live with [43].

The current study reported that the number of patients testing positive for COVID-19 has exponentially risen, exerting pressure on the limited public health facilities and health personnel in the country. Also, the fear of incapacity to handle COVID-19 patients had elicited anxiety among healthcare workers, due to the nature and novelty of the virus. These findings agree with studies undertaken in China during the onset of the pandemic [10, 14].

In the current study, parameter estimates revealed that for every unit increase in the level of anxiety, male respondents were 0.855 times less likely to experience anxiety as a result of COVID-19 than females, similar to anxiety, female healthcare workers were more likely to be depressed as a result of the COVID-19 disease pandemic, and this agrees with another study which was done during epidemics of similar nature [44]. The observed differences can be attributed to gender-based roles for women at the household level. Available studies have also noted that anxiety symptoms positively predict later depressive symptoms [45, 46] and therefore there is a need to put measures in place once there is an indication of anxiety as this will greatly influence the development of depressive symptoms [47].

Also, the current study reported a relatively high number of frontier healthcare workers without confidence in managing COVID-19 cases. This would be attributed to the novelty of the disease. This finding is in agreement with other studies conducted during disease outbreaks and pandemics in different regions [48, 49], which have indicated that during the onset of pandemics, the healthcare workers are generally not well prepared especially in instances where the outbreaks are novel.

## 5 Conclusion

The findings of the current study indicates that the majority of healthcare workers had mild anxiety and depression. Female healthcare workers were more likely to experience severe anxiety and depression and the levels of anxiety and depression differed across different cadres of respondents. Therefore, there is a need for psychosocial support for frontline healthcare workers during the pandemics.

### 5.1 Strengths and limitations

The study utilized validated tools and methodologies in assessing the levels of anxiety and depression. Whereas the data was generally representative of the study population, the scope of the current study was limited to assessing anxiety and depression levels among frontier healthcare workers due to the novel 2019 coronaviruses disease.

## Declarations

### Author contribution statement

David Onchonga: Conceived and designed the experiments; Performed the experiments; Analyzed and interpreted the data; Wrote the paper.

Enoch Ngetich, Diana Wangeshi: Performed the experiments; Analyzed and interpreted the data.

Wilbroda Makunda, Pius Wainaina: Analyzed and interpreted the data; Wrote the paper.

Viktoria Prémusz: Contributed reagents, materials, analysis tools or data; Wrote the paper.

### Funding statement

This research did not receive any specific grant from funding agencies in the public, commercial, or not-for-profit sectors.

### Data availability statement

Data will be made available on request.

### Declaration of interests statement

The authors declare no conflict of interest.

### Additional information

No additional information is available for this paper.
